# A genomics approach identifies senescence-specific gene expression regulation

**DOI:** 10.1111/acel.12234

**Published:** 2014-05-23

**Authors:** Daniel H Lackner, Makoto T Hayashi, Anthony J Cesare, Jan Karlseder

**Affiliations:** Salk Institute for Biological Studies, Molecular and Cell Biology Laboratory10010 North Torrey Pines Road, La Jolla, CA, 92037, USA; * Children’s Medical Research Institute, Genome Integrity Group214 Hawkesbury Rd., Westmead, NSW, 2145, Australia

**Keywords:** replicative aging, senescence, DNA damage, telomerase expression, cell cycle

## Abstract

Replicative senescence is a fundamental tumor-suppressive mechanism triggered by telomere erosion that results in a permanent cell cycle arrest. To understand the impact of telomere shortening on gene expression, we analyzed the transcriptome of diploid human fibroblasts as they progressed toward and entered into senescence. We distinguished novel transcription regulation due to replicative senescence by comparing senescence-specific expression profiles to profiles from cells arrested by DNA damage or serum starvation. Only a small specific subset of genes was identified that was truly senescence-regulated and changes in gene expression were exacerbated from presenescent to senescent cells. The majority of gene expression regulation in replicative senescence was shown to occur due to telomere shortening, as exogenous telomerase activity reverted most of these changes.

## Introduction, results, discussion

Human somatic cells do not have the ability to divide indefinitely (Hayflick & Moorhead, 1961) but will eventually enter replicative senescence, triggered by genomic stress, most of which is attributed to telomere shortening (Campisi & d’Adda di Fagagna, 2007). Failure to initiate senescence has detrimental effects and results in the emergence of transformed and immortalized cells (Artandi & DePinho, 2000). Human fibroblasts such as the normal diploid fibroblasts cell line IMR90 are the major model system to study replicative senescence (Shay *et al*., 1991; Narita *et al*., 2003). Here, we examined global gene expression by analyzing a time-course as IMR90 cells progressed toward senescence. We show that re-introduction of telomerase into presenescent cells reverted the majority of gene expression changes, and we compared these data with changes in response to DNA damage and quiescence. This work represents a resource for the field of senescence and aging research.

**Figure 1:**
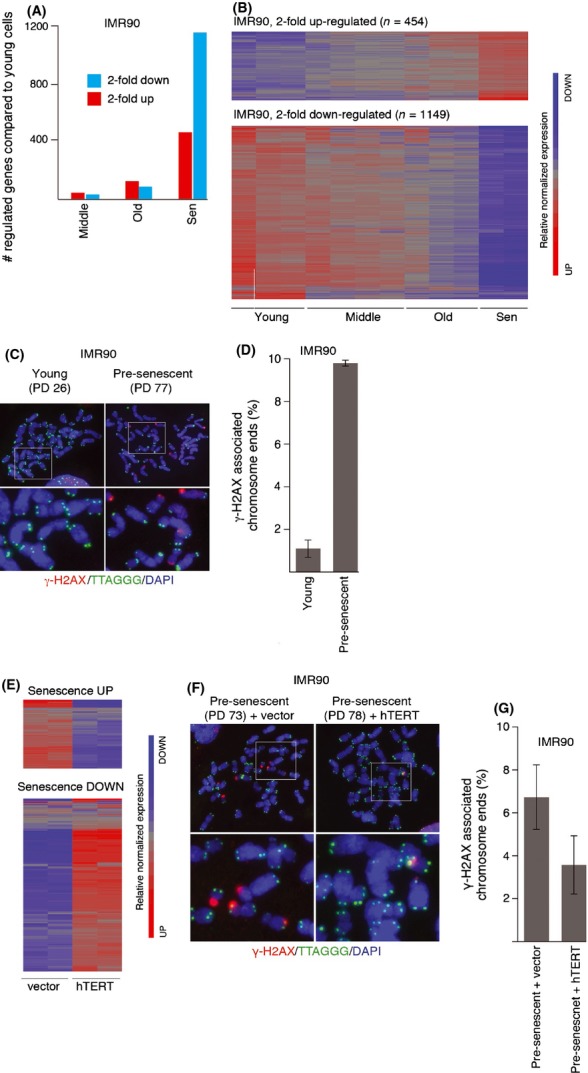
Gene expression regulation in response to senescence is reverted upon hTERT expression. (A) Number of twofold regulated genes compared to young cells for the indicated time points. (B) Hierarchical clustering of twofold regulated genes in senescent IMR90 cells compared to young cells. Relative expression values are indicated for: young cells (PD 30) 3 repeats, middle cells (PD 50) four repeats, old cells (PD 70) three repeats and senescent cells (PD 80) 2 repeats. (C) TIF analysis: γH2AX colocalization events in young and presenescent cells. Green signal: telomere specific FISH probe, red signal: DNA damage marker γH2AX. (D) Quanification of (C). (E) Hierarchical clustering of senescence-regulated genes [same as in (A)] according to gene expression values in presenescent cells expressing hTERT or vector control. Relative expression values are indicated. (F) TIF analysis: γH2AX colocalization events in presenescent cells expressing hTERT or vector control. Green signal: telomere specific FISH probe, red signal: γH2AX. (G) Quantification of (F).

To describe global senescence-associated expression patterns, we grew IMR90 cells at physiological oxygen levels from low population doublings (PD) onward until they reached replicative senescence and analyzed RNA levels with Affymetrix arrays. Genes were regulated as a function of replicative age, as the number of regulated genes at each time point increased (Fig. 1A), with 1603 genes regulated in senescent cells (Table S1). Expression changes were exacerbated with a progression toward senescence, pointing toward regulation of specific pathways (Fig. 1B). Array data for selected genes were confirmed in IMR90 and WI38 cells (Fig. S1A,B). We confirmed the impact of replicative senescence on telomeres by assessing the number of telomere dysfunction-induced foci (TIF) (Takai *et al*., 2003). While young cells displayed only background levels, TIF were increased in presenescent cells (Fig. 1C,D), which is in agreement with previous reports (Kaul *et al*., 2012).

We next determined enriched pathways. Down-regulated genes were strongly enriched for proliferation and replication pathways, while the pathways associated with genes that were up-regulated were less congruent, but there was enrichment for pathways that impact on proliferation (Table S2), for example TGFβ signaling, which can repress proliferation (Hannon & Beach, 1994). We also identified genes with established roles in senescence (p21, p16) or with antiproliferative properties such as WNT16 (Binet *et al*., 2009), or BTG2 (Rouault *et al*., 1996). Enrichment of these pathways together with the identification of previously reported genes confirmed the reproducibility of our data.

Next, we assessed the impact of hTERT on gene expression in senescence. We generated presenescent cells expressing hTERT and confirmed expression and telomere elongation (Fig. S2). Most expression changes in senescence were reverted upon hTERT expression (Fig. 1E). Eighty-four percent of the initially up-regulated and 86% of the down-regulated genes showed at least a 1.2-fold reversion. While expression was not in all cases completely restored, only few genes failed to show any reversion. Hierarchical clustering also pointed out a reversion (Fig. S3): Expression profiles from initially presenescent cells expressing hTERT were more similar to young cells than to senescent cells.

Why were few genes (Table S3) not reverted upon hTERT expression? 52 of 55 down-regulated genes showed already at least a 1.2-fold change between young and middle cells, and only 9 of these displayed an additional change in senescence arguing against regulation due to senescence. Also, genes such as p16 contribute to senescence in a telomerase-independent way (Herbig *et al*., 2004). Alternatively, timing of expression might be important. Cells were infected with hTERT or control constructs at PD 65 (old) and were presenescent at the time of harvesting. Consequently, p16 was not yet increased to the level of senescent cells, and suppression was thus not observed (Fig. S4).

We ruled out that hTERT itself had an effect on gene expression, as it was recently shown that TERT can modulate transcription through direct association with chromatin (Choi *et al*., 2008). Expression of hTERT in young IMR90 cells that still possess a reservoir of long telomeres had no effect on gene expression, with only hTERT itself showing a significant change (Fig. S2). Examination of the number of TIF in presenescent cells indicated a significant reduction following the expression of hTERT (Fig. 1F,G). These data suggest that the majority of gene expression changes in senescent cells happened as consequence of telomere shortening and DNA damage signaling and that hTERT-mediated reversion of gene expression changes was due to telomere elongation and suppression of DNA damage.

**Figure 2:**
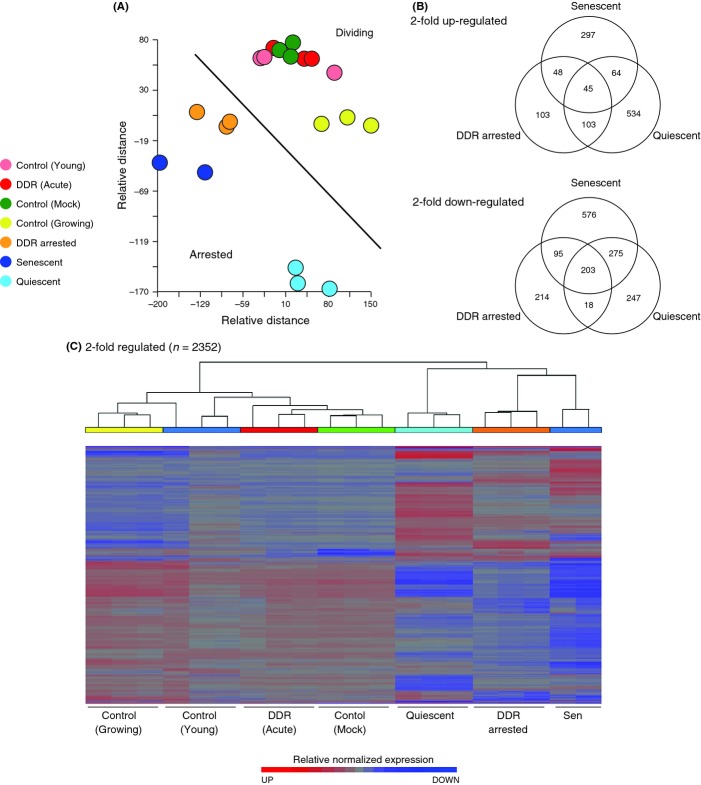
Similar gene expression profiles in cells arrested in senescence, quiescence and in response to DNA damage by IR. (A) PCA of all arrays shows a distinction in overall gene expression phenotypes between cycling and arrested cells. (B) Overlap of regulated genes in senescence, quiescence or in response to DNA damage (DDR arrested). All overlaps are statistically significant (p-values <0.05). (C) Hierarchical clustering of regulated genes in either senescence, quiescence, acute DDR, or in cells arrested after DDR.

There was strong enrichment for cell cycle and proliferation pathways within the senescence-regulated genes (Table S1). Consequently, we asked to what extent expression regulation in senescence simply reflects changes due to cell cycle arrest and if we can identify senescence-specific pathways that are not directly linked to the cell cycle. We irradiated cells with 5 Gy of ionizing radiation (IR) and analyzed expression 30 min after and when cells were permanently arrested 5 days later. Additionally, we analyzed gene expression patterns of quiescent IMR90 cells, arrested by serum starvation. Principle component analysis (PCA) showed that there was a distinction between gene expression profiles from cycling and arrested cells (Fig. 2A): control conditions and expression profiles from the 30 min time point after IR clustered together, while profiles from senescent, quiescent, and IR-arrested cells clustered together. 30 min post-IR, no genes showed a significant down-regulation, and only three genes showed a significant up-regulation: GDF15, BTG2, and p21, all direct p53 targets with established antiproliferative roles (Rouault *et al*., 1996; Agarwal *et al*., 2006; Sperka *et al*., 2012).

The number of genes regulated in quiescent and IR-arrested cells was similar to the number regulated in senescence, with overlap between regulated genes (Fig. 2B). While not all genes showed a twofold regulation in all conditions, the general trend went in the same direction, determined by hierarchical clustering (Fig. 2C), which recapitulated the PCA. These data demonstrate that the majority of expression changes in senescent cells was due to a stop in proliferation and cell cycle arrest and was similar to other conditions that halt proliferation.

Lastly, we determined genes that exhibited senescence-specific regulation and were not regulated as a response to DNA damage or quiescence (for selection criteria see Experimental procedures) (Table S4). Few pathways showed enrichment for regulated genes (Table S5), and only few genes were responsible for the enrichment. There was enrichment for regulators of cytokine signaling, due to down-regulation of SOCS1, SOCS3, and LIFR, all of which have a reported role in negative regulation of cytokine signaling, consistent with an increase in cytokine signaling in senescent cells (SASP) (Coppé *et al*., 2008). Analyzing gene ontology (GO) terms, we found a strong enrichment for proteins that are associated with the cellular membrane (Table S5), in agreement with altered morphology and increased adhesion of senescent cells to the extracellular matrix, usually mediated through membrane-associated proteins.

We also focused on RFPL4A, which was specifically up-regulated in replicative senescence (Fig. S1A-C, Table S4). Our qPCR data (Fig. S1A,B) suggest that RFPL4A was regulated in an on-off fashion, as it was barely detectable in young cells. RFPL4A is a putative ubiquitin-ligase and has been shown to target cyclin B1 for degradation (Suzumori *et al*., 2003). We confirmed up-regulation in replicative senescence at the protein level (Fig. S1C), but over-expression of RFPL4A in young IMR90 cells had no effect on proliferation or cell cycle progression. Still, RFPL4A is a potential novel senescence marker.

Here, we described a novel and unique approach to senescence-associated expression changes in human IMR90 fibroblasts: Most importantly, by not only comparing endpoints (young and senescent cells), but describing gene expression changes in cells as they progressed toward senescence and the reversion of these changes upon hTERT expression, we were able to generate a high-quality curated dataset that reflects the dynamic content of the path to senescence.

We also demonstrated that there are only few pathways that are uniquely engaged during replicative senescence and that the majority of regulated genes is commonly altered in cells that are cell cycle arrested due to other triggers, such as DNA damage or serum starvation. Together with our data on hTERT re-introduction, this confirms that replicative senescence is not a completely autonomous program, but a specialized cell cycle arrest that occurs in response to nonrepairable DNA damage, mostly at telomeric regions.

Global gene expression regulation during senescence has been the topic of previous studies, but the combination of analysis presented in our study is unique, especially as many published studies either use oncogene-induced senescence (Chicas *et al*., 2010; Aksoy *et al*., 2012) as model for senescence or grow cells at atmospheric oxygen levels (Kim *et al*., 2013), both of which dramatically alter gene expression programs.

In conclusion, we provide the first study to describe gene expression changes in replicative senescence in IMR90 fibroblasts grown at physiological oxygen levels. Our data provide a framework and novel potential candidates for future studies to identify truly senescence-specific genes and their involvement in establishing and maintaining a permanent cell cycle arrest.

## Supporting Information

**Fig. S1.** Quantitative RT–PCR data, protein levels of RFPL4A.

**Fig. S2.** hTERT expression and telomere length analysis.

**Fig. S3.** Expression levels p21, p16.

**Fig. S4.** Different timing in gene expression regulation during senescence.

**Table S1.** List of all senescence-regulated genes.

**Table S2.** Enriched pathways within all senescence-regulated genes.

**Table S3.** List of senescence-regulated genes that are not reverted upon hTERT expression.

**Table S4.** List of senescence-specific regulated genes.

**Table S5.** Enriched pathways within senescence-specific regulated genes

**Table S6.** Primers used for qPCR analysis.

**Data S1.** Materials and Methods, Accession numbers.
