# Supplementary material for: A genomics approach identifies senescence-specific gene expression regulation
Source: Aging Cell. 2014 May 23;13(5):946–50. doi: 10.1111/acel.12234 (PMC4172521; doi:10.1111/acel.12234)
Supplement: Supplementary file 1 — Fig. S1. Quantitative RT–PCR data, protein levels of RFPL4A. [file acel0013-0946-sd1.pdf]

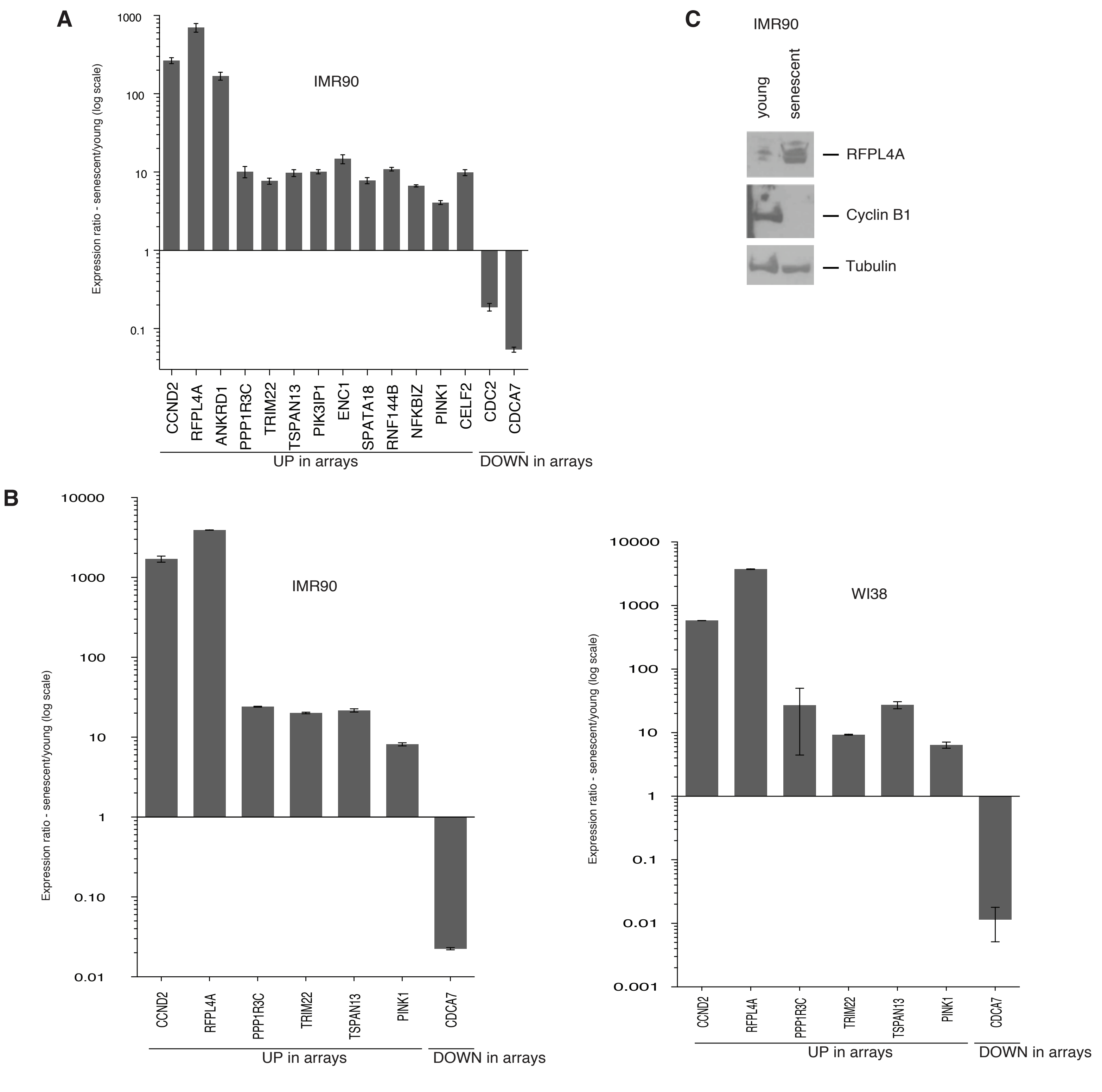

Fig. S1. **(A)** Quantitative RT-PCR data from 4 repeats showing expression ratios for indicated mRNAs comparing senescent to young IMR90 cells. **(B)** Quantitative RT-PCR data from 2 repeats showing expression ratios for indicated mRNAs comparing senescent to young cells in 2 additional experiments for 2 cell lines (IMR90 and WI38). **(C)** Western blot showing protein levels of RFPL4A in young and senescent IMR90 cells. Cyclin B1 and γ-Tubulin levels are shown as control. Antibodies used: monoclonal anti-γ-Tubulin (Sigma-Aldrich, T6557), polyclonal cyclin B1 (Santa Cruz Biotechnology, sc-752), polyclonal RFPL4A (Santa Cruz Biotechnology, sc-169141)
