# Supplementary material for: A genomics approach identifies senescence-specific gene expression regulation
Source: Aging Cell. 2014 May 23;13(5):946–50. doi: 10.1111/acel.12234 (PMC4172521; doi:10.1111/acel.12234)
Supplement: Supplementary file 2 — Fig. S2. hTERT expression and telomere length analysis. [file acel0013-0946-sd2.pdf]

A

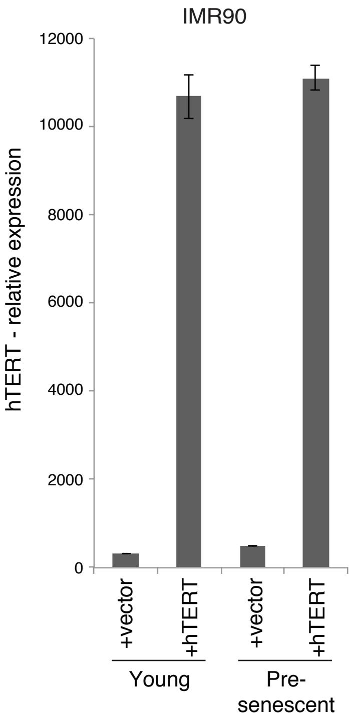

B

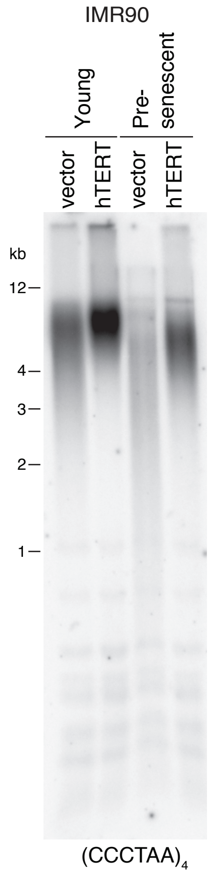

Fig. S2. (A) Relative gene expression of the hTERT gene as measured using Affymetrix arrays in young and pre-senescent IMR90 cells. **(B)** Telomeric restriction fragment analysis (TRF) by southern blotting of AluI/MboI-digested genomic DNA with a telomere-specific probe.
