# Supplementary material for: A genomics approach identifies senescence-specific gene expression regulation
Source: Aging Cell. 2014 May 23;13(5):946–50. doi: 10.1111/acel.12234 (PMC4172521; doi:10.1111/acel.12234)
Supplement: Supplementary file 3 — Fig. S3. Expression levels p21, p16. [file acel0013-0946-sd3.pdf]

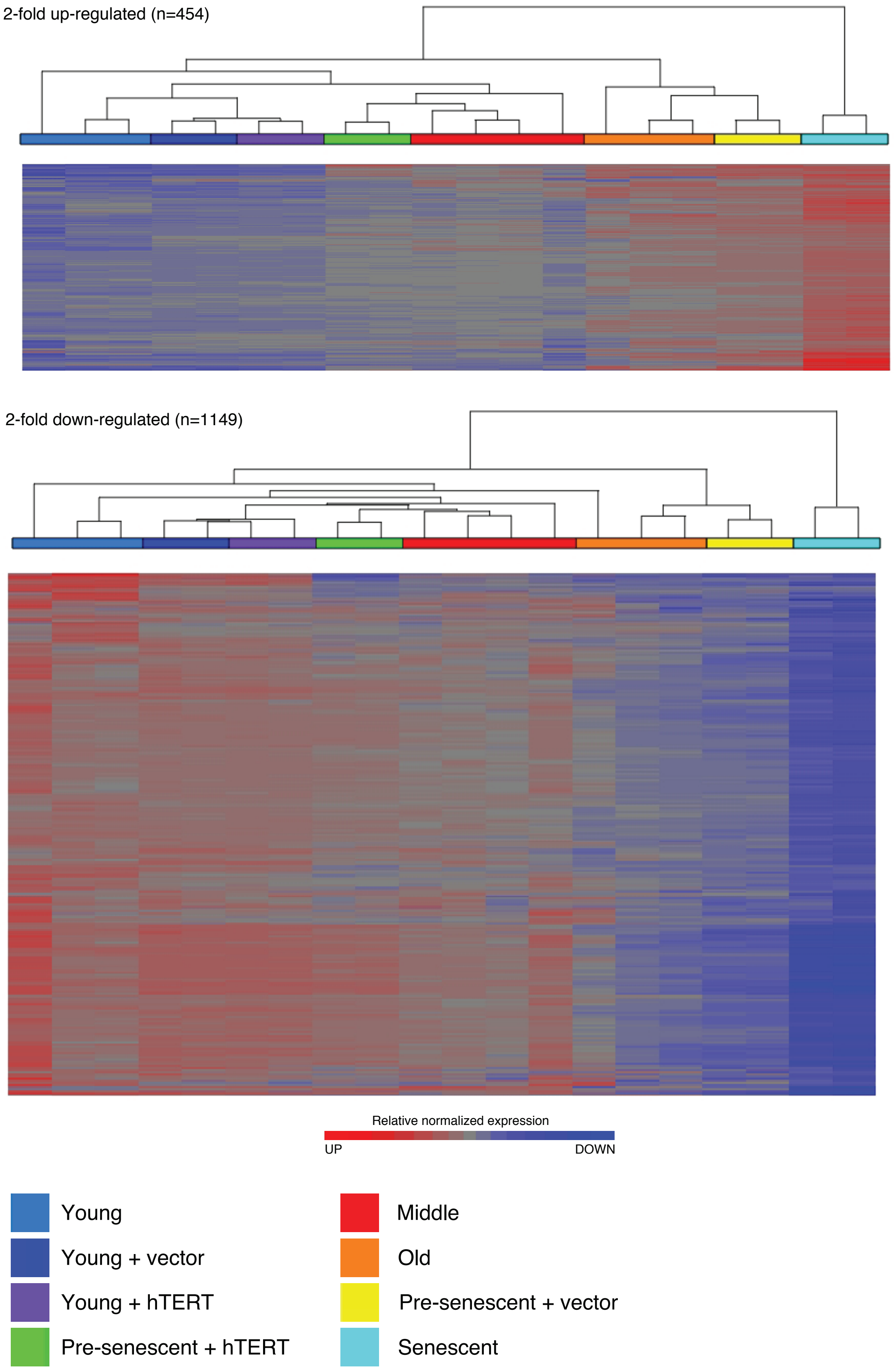

Fig. S3. Reversion of senescence-specific expression regulation upon re-introduction of hTERT. Unbiased hierarchical clustering for all genes that are 2-fold up- or down-regulated in senescent cells compared to young cells for the indicated conditions.
