# Supplementary material for: A genomics approach identifies senescence-specific gene expression regulation
Source: Aging Cell. 2014 May 23;13(5):946–50. doi: 10.1111/acel.12234 (PMC4172521; doi:10.1111/acel.12234)
Supplement: Supplementary file 4 — Fig. S4. Different timing in gene expression regulation during senescence. [file acel0013-0946-sd4.pdf]

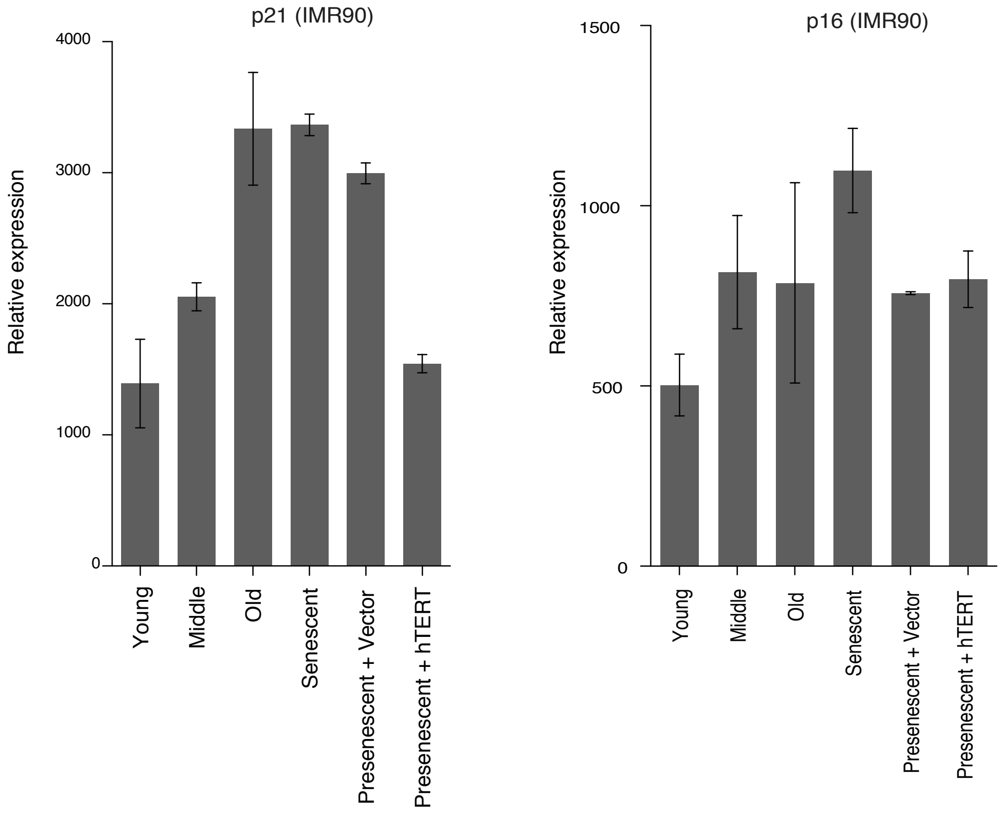

Fig. S4. Different timing in gene expression regulation during senescence. Relative gene expression of p21 and p16 in IMR90 cells as measured using Affymetrix arrays for the indicated time points.
