# Supplementary material for: A genomics approach identifies senescence-specific gene expression regulation
Source: Aging Cell. 2014 May 23;13(5):946–50. doi: 10.1111/acel.12234 (PMC4172521; doi:10.1111/acel.12234)
Supplement: Supplementary file 6 — Table S2. Enriched pathways within all senescence-regulated genes. [file acel0013-0946-sd6.doc]

**Table S2. Enriched pathways within all senescence-regulated genes**

| **Down-regulated genes** |  |
| --- | --- |
| **Signal Transduction Pathways (canonical)** | **P value** |
| PLK1 signaling events | 3.01E-16 |
| Aurora B signaling | 4.28E-14 |
| ATR signaling pathway | 6.77E-13 |
| E2F transcription factor network | 1.09E-10 |
| cdk regulation of dna replication | 3.46E-10 |
| BARD1 signaling events | 1.20E-09 |
| brca1 dependent ub ligase activity | 1.32E-08 |
| role of brca1 brca2 and atr in cancer susceptibility | 1.01E-07 |
| FOXM1 transcription factor network | 2.62E-07 |
| ATM pathway | 2.63E-06 |
| Aurora A signaling | 3.53E-05 |
| cyclins and cell cycle regulation | 1.96E-04 |
| role of ran in mitotic spindle regulation | 1.98E-04 |
| cell cycle: g1/s check point | 4.14E-03 |
| rb tumor suppressor/checkpoint signaling in response to dna damage | 4.85E-03 |
| cell cycle: g2/m checkpoint | 6.76E-03 |
| Aurora C signaling | 9.14E-03 |
|  |  |
| **Signal Transduction Pathways (Genomatix Literature Mining)** | **P value** |
| CHECKPOINT | 5.37E-39 |
| CELL DIVISION CYCLE 2, G1 TO S AND G2 TO M | 3.95E-30 |
| POLO LIKE KINASE 1 | 8.20E-30 |
| CELL CYCLE | 5.17E-27 |
| DNA REPAIR | 5.21E-27 |
| CHK1 CHECKPOINT HOMOLOG | 9.89E-26 |
| CYCLIN A2 | 8.46E-19 |
| AURORA KINASE | 1.68E-17 |
| CYCLIN B1 | 3.26E-17 |
| FANCONI ANEMIA COMPLEMENTATION GROUP COMPLEX | 1.17E-15 |
| CYCLIN DEPENDENT KINASE | 7.37E-15 |
| ATAXIA TELANGIECTASIA AND RAD3 RELATED | 3.01E-14 |
| BREAST CANCER 1, EARLY ONSET | 3.71E-13 |
| ATAXIA TELANGIECTASIA MUTATED | 2.20E-12 |
| BREAST CANCER 2, EARLY ONSET | 5.16E-12 |
| MINICHROMOSOME MAINTENANCE COMPLEX | 1.03E-11 |
| WEE1 HOMOLOG | 4.57E-09 |
| CELL DIVISION CYCLE 25C | 1.77E-08 |
| TUMOR PROTEIN P53 | 2.91E-08 |
| DNA DEPENDENT PROTEIN KINASE | 8.24E-07 |
| CYCLIN DEPENDENT KINASE INHIBITOR 1 | 2.22E-06 |
| RETINOBLASTOMA 1 | 4.48E-06 |
| CYCLIN E | 2.04E-05 |
| CYCLIN D1 | 5.30E-05 |
| E2F TRANSCRIPTION FACTOR 1 | 2.41E-04 |
| NIMA (NEVER IN MITOSIS GENE A) RELATED KINASE | 2.71E-04 |
| CYCLIN B2 | 3.71E-04 |
| RAN, MEMBER RAS ONCOGENE FAMILY | 9.06E-04 |
| PROTEIN PHOSPHATASE 2 | 2.01E-03 |
| CYCLIN D3 | 3.42E-03 |
| SMOOTHENED HOMOLOG (DROSOPHILA) | 6.55E-03 |
| PROTEOLYTIC | 7.48E-03 |
| BACULOVIRAL IAP REPEAT CONTAINING PROTEIN, APOPTOSIS INHIBITOR | 7.85E-03 |
| PATCHED HOMOLOG 1 (DROSOPHILA) | 8.27E-03 |
| PHOSPHATASE AND TENSIN HOMOLOG | 9.43E-03 |
|  |  |
|  |  |
| **Up-regulated genes** |  |
| **Signal Transduction Pathways (canonical)** | **P value** |
| Glypican 1 network | 2.10E-03 |
| Beta1 integrin cell surface interactions | 4.12E-03 |
| agrin in postsynaptic differentiation | 4.47E-03 |
| Cytosolic calcium ion concentration elevation (through IP3 receptor) ( GPCR signaling (G alpha q) ) | 9.17E-03 |
|  |  |
| **Signal Transduction Pathways (Genomatix Literature Mining)** | **P value** |
| ANGIOGENESIS | 2.95E-04 |
| MATRIX METALLOPROTEINASE | 6.73E-04 |
| CADHERIN 5, TYPE 2 (VASCULAR ENDOTHELIUM) | 1.07E-03 |
| PAIRED LIKE HOMEODOMAIN 2 | 2.19E-03 |
| TGF BETA | 3.23E-03 |
| LOW DENSITY LIPOPROTEIN RECEPTOR RELATED PROTEIN | 5.76E-03 |
| NF KAPPA B | 8.63E-03 |

Significantly enriched signal transduction pathways (canonical and derived from literature mining) within genes that are either 2-fold up- or down-regulated in senescent cells compared to young cells as determined using the Genomatix software.
