# Supplementary material for: A genomics approach identifies senescence-specific gene expression regulation
Source: Aging Cell. 2014 May 23;13(5):946–50. doi: 10.1111/acel.12234 (PMC4172521; doi:10.1111/acel.12234)
Supplement: Supplementary file 9 — Table S5. Enriched pathways within senescence-specific regulated genes [file acel0013-0946-sd9.doc]

**Table S5. Enriched pathways within senescence-specific regulated genes**

| **Down-regulated genes** |  |
| --- | --- |
| **Signal Transduction Pathways (canonical)** | **P-value** |
| Growth hormone signaling pathway (JAK2 STAT5) | 8.71E-04 |
|  |  |
| **Signal Transduction Pathways (Genomatix Literature Mining)** | **P-value** |
| GRANULOCYTE COLONY STIMULATING FACTOR | 3.99E-04 |
| DEVELOPMENTAL | 6.56E-04 |
| LEUKEMIA INHIBITORY FACTOR | 7.61E-04 |
| GROWTH HORMONE RECEPTOR | 2.75E-03 |
| INTERLEUKIN 6 SIGNAL TRANSDUCER (GP130, ONCOSTATIN M RECEPTOR) | 5.79E-03 |
| CILIARY NEUROTROPHIC FACTOR RECEPTOR | 5.79E-03 |
| INTERLEUKIN 6 (INTERFERON, BETA 2) | 9.98E-03 |
|  |  |
| **GO-Term (Molecular function)** | **P-value** |
| latrotoxin receptor activity | 6.78E-03 |
| leukemia inhibitory factor receptor activity | 6.78E-03 |
| protein kinase inhibitor activity | 7.76E-03 |
| collagen binding | 8.15E-03 |
| kinase inhibitor activity | 8.55E-03 |
| carbohydrate binding | 9.22E-03 |
|  |  |
| **GO-Term (Cellular Component)** | **P-value** |
| extracellular matrix | 2.81E-03 |
| Bcl3/NF-kappaB2 complex | 7.56E-03 |
| guanylate cyclase complex, soluble | 7.56E-03 |
|  |  |
|  |  |
| **Up-regulated genes** |  |
| **Signal Transduction Pathways (canonical)** | **P-value** |
| a6b1 and a6b4 Integrin signaling | 8.76E-03 |
|  |  |
| **GO-Term (Molecular function)** | **P-value** |
| calcium-dependent phospholipid binding | 5.83E-03 |
| 1-alkylglycerophosphocholine O-acetyltransferase activity | 8.47E-03 |
| alpha-L-fucosidase activity | 8.47E-03 |
| natural killer cell lectin-like receptor binding | 8.47E-03 |
| alpha-2C adrenergic receptor binding | 8.47E-03 |
| follistatin binding | 8.47E-03 |
| fucosidase activity | 8.47E-03 |
|  |  |
| **GO-Term (Cellular Component)** | **P-value** |
| membrane | 1.73E-04 |
| integral to membrane | 2.52E-04 |
| membrane part | 3.15E-04 |
| intrinsic to membrane | 3.90E-04 |
| apical part of cell | 2.06E-03 |
| basement membrane | 3.20E-03 |
| Golgi membrane | 3.38E-03 |
| apical plasma membrane | 5.63E-03 |
| plasma membrane | 7.73E-03 |
| organelle membrane | 7.76E-03 |
| inhibin A complex | 8.16E-03 |
| laminin-2 complex | 8.16E-03 |
| inhibin complex | 8.16E-03 |
| laminin-5 complex | 8.16E-03 |
| cell periphery | 9.36E-03 |
| Golgi apparatus part | 9.48E-03 |

Significantly enriched signal transduction pathways and gene ontology (GO) terms within genes that are specifically regulated in senescent cells, but show no regulation in quiescent cells or in cells arrested in in response to IR.
