# Supplementary material for: A genomics approach identifies senescence-specific gene expression regulation
Source: Aging Cell. 2014 May 23;13(5):946–50. doi: 10.1111/acel.12234 (PMC4172521; doi:10.1111/acel.12234)
Supplement: Supplementary file 11 — Data S1. Materials and Methods, Accession numbers. [file acel0013-0946-sd11.doc]

**Experimental procedures**

**Cell lines, culture conditions, senescence time course**

All experiments were performed using IMR90 and WI38 cells, which were grown in DMEM medium containing 15% Fetal Bovine Serum, antibiotics (Penicillin, Streptomycin), and non-essential amino acids. Phoenix-Ampho packaging cells were grown in 10% Fetal Bovine Serum. Cells were grown at 7.5% CO2 and 3% O2. Before infections cells were grown in the absence of antibiotics. We prepared RNA samples from young (PD 30), middle (PD 50) and old (PD 70) cells, all of which were still dividing at comparable rates. Additionally, we prepared RNA samples from senescent cells (PD 80), which had stopped dividing, as indicated by a lack of increase in cell numbers even after 7 days of incubation. RNA samples were prepared from the indicated time points (2-4 biological repeats), labeled and hybridized to Affymetrix Gene 1.0 ST arrays.

**Plasmids and transfections**

Phoenix-Ampho cell transfection with pBABE-puro or pBABE-puro-hTERT and subsequent infection with viral supernatants and selection of IMR90 was done as described (Crabbe et al. 2004).

**RNA extraction, Affymetrix array hybridization and analysis**

RNA was extracted using Trizol (Invitrogen) and further purified using RNeasy mini kits (Qiagen). Isolated RNA (100 ng) was used for generation of cDNA and terminal labelling using the Ambion® WT Expression Kit and GeneChip® WT Terminal Labeling and Controls Kit and labeled probes were hybridized to Affymetrix GeneChip® Human Gene 1.0 ST Arrays. Arrays were scanned on a GeneChip Scanner 3000 7G. Cell files were normalized using the Partek Genomics Suite (RMA background correction, quantile normalization, median polishing). Differentially expressed genes were detected using the ANOVA function in the Partek Genomics Suite. To define differentially expressed genes, we used a 2-fold cutoff and a p-value adjusted for false discovery rate of <0.05. Hierarchical clustering and PCA analysis was performed using the Partek Genomics Suite and expression values for the clusters were standardized by shifting the mean to zero. Enriched functional pathways were defined using the Generanker tool in the Genomatix Genome Analyzer software (<http://www.genomatix.de/solutions/genomatix-genome-analyzer.html>).

P-values for the overlap of gene lists were calculated using the webtool at <http://nemates.org/MA/progs/overlap_stats.html>.

**qPCR analyis**

For qPCR analysis, cDNA was generated from 1 ug of RNA using the QuantiTect Reverse Transcription Kit (Qiagen). 25 ul of H_2_O was added to the resulting 20 ul of cDNA and 2.5 ul of this cDNA mix was used for each qPCR reaction with SYBR green on a Bio-Rad CFX96 Real-Time PCR Detection System. Expression values for the corresponding genes were normalized to actin (ACTB) mRNA. For all primer sequences see Table S6.

**Filtering for senescence-specific gene regulation**

We chose genes that were 2-fold regulated in senescence, but did not show more than a 1.2-fold regulation in both quiescent and DDR-arrested cells. We further removed genes that showed an inverse regulation of 1.2-fold in middle or old cells and genes that were already 2-fold regulated in middle cells. Using these criteria we removed 16 and 21 genes from the up- and down-regulated list, respectively, and ended up with a list of 94 up- and 85 down-regulated senescence-specific genes (Table S4).

**Meta-TIF assay**

The Meta-TIF assay was performed as described previously (Cesare et al. 2013).

**Telomere length analysis**

Genomic DNA preparation and TRF analysis were done as described previously (Karlseder et al. 2002).

**Accession numbers**

Accession numbers are as follows: E-MTAB-2086, senescence time course; E-MTAB-2087, hTERT expression; E-MTAB-2088, DNA damage; E-MTAB-2105, quiescence.

**Additional References**

Cesare AJ, Hayashi MT, Crabbe L & Karlseder J (2013) The telomere deprotection response is functionally distinct from the genomic DNA damage response. *Mol. Cell* 51, 141–155.

Crabbe L, Verdun RE, Haggblom CI & Karlseder J (2004) Defective telomere lagging strand synthesis in cells lacking WRN helicase activity. *Science* 306, 1951–1953.

Karlseder J, Smogorzewska A & de Lange T (2002) Senescence induced by altered telomere state, not telomere loss. *Science* 295, 2446–2449.
